# Identification of Genes Accurately Predicting Fiber Length for AI-Powered Gene-Based Breeding Across Populations and Environments in Cotton

**DOI:** 10.3390/ijms27198908

**Published:** 2026-10-07

**Authors:** Yuan Ye, Yun-Hua Liu, C. Wayne Smith, Sing-Hoi Sze, Hong-Bin Zhang

**Affiliations:** 1 Department of Soil and Crop Sciences, Texas A&M AgriLife Research and Texas A&M University, College Station, TX 77843, USA; 2 Departments of Computer Science and Engineering and Biochemistry and Biophysics, Texas A&M University, College Station, TX 77843, USA

**Keywords:** gene-based breeding, gene, fiber length, functional SNPs, prediction accuracy, cotton

## Abstract

AI-powered gene-based breeding (AI-GBB) has the potential to be a next-generation breeding technology. However, it is unclear whether agronomic genes identified with one population can be reliably used for AI-GBB across diverse populations, environments, and eco-agriculture systems. We evaluated 474 cotton fiber length (*GFL*) genes using two diverse populations grown across 11 environments in China and the United States. Four hundred and seventy-five functional SNPs significantly impacting fiber length were identified from 143 of the genes and tested for AI-GBB. Either the 475 functional SNPs or their 143 SNPs with one SNP per gene predicted fiber lengths across environments using either the total number of their favorable alleles or themselves as DNA markers at accuracies of 0.75–0.89, outperforming genomic selection by several times. The prediction model trained in one environment accurately and consistently predicted fiber length in all other environments. We found that the *GFL* genes enriched in proteolysis, protein binding, and UDP-sugar pyro-phosphorylases play important roles in their predictability. Our results demonstrate that agronomic genes identified from one population can be robust predictors of quantitative traits across diverse populations and environments for AI-GBB. The functional SNPs provide the most desirable tools for enhanced cotton breeding with all molecular breeding methods developed to date, and the utility knowledge of the genes is necessary for the development and deployment of AI-GBB in all crops.

## 1. Introduction

The research, development, and application of new or advanced technologies are crucial to enhanced and sustained crop genetic improvement, and thus agriculture production. The recently developed artificial-intelligence-powered gene-based breeding (AI-GBB) has the potential to be a revolutionary technology for predictable, rapid, high-throughput, and cost-efficient development of superior varieties for agriculture production [1,2]. Unlike current genomic selection- or genomics-assisted breeding that conducts parent selection and cross-design largely based on the phenotypes of objective and related agronomic traits and performs progeny selection using the phenotypes of targeted traits predicted using genome-wide random omic features, such as SNPs, AI-GBB enables breeders to develop superior varieties applicable to different environments and/or eco-agriculture systems worldwide for agriculture production with a computer in a factory-production-like manner. AI-GBB allows breeders to develop superior varieties by design, based on the contents of the genes controlling breeding objectives and related agronomic traits (hereafter, agronomic genes). This is powered by AI throughout the entire breeding process, including parent selection, cross-design, and progeny selection, before multi-location variety testing. Studies have shown that AI-GBB can be hundreds of times more efficient and productive, 5–8 years or 50–80% faster, and hundreds of times more cost-efficient than current genomic-selection- or genomics-assisted breeding methods [1,2,3,4,5,6]. Liu et al. [7] showed that AI-GBB could potentially further improve the current best varieties by an additional 73–118%.

AI-GBB is developed following three steps [1,2]. First, most, if not all, genes controlling yield and agronomic traits are identified for a target crop species and AI-GBB is initiated while the utility and efficiency of the agronomic genes for AI-GBB are verified. Then, most, if not all, cultivars, breeding lines and/or germplasm accessions used in different breeding programs of the crop—either nationwide or worldwide—are genotyped with the yield and agronomic genes. Finally, a database of yields and agronomic genes of the cultivars, breeding lines, and germplasm accessions is constructed for AI-GBB (the AI-GBB database). Because the genetic variations in yield and most, if not all, of agronomic traits, result from variations in their controlling genes even though they may be modulated by epigenetic factors and/or genotype-by-environment (G × E) interactions, the AI-GBB database consists of agronomic genes and their variants in the cultivars, breeding lines, and germplasm accessions used in different breeding programs of the crop, such as single-nucleotide polymorphisms (SNPs), nucleotide insertion/deletions (InDels), transcript splices and expressions, and the genetic effects of the yield and agronomic genes, such as favorable and unfavorable alleles and complete or over-dominant genotypes.

AI-GBB, once initiated, can be immediately used to develop superior varieties for production, though its robustness and efficiency is dependent on the robustness of the AI-GBB database, including the numbers of yield and agronomic genes, and the numbers of cultivars, breeding lines, and germplasm accessions genotyped with the yield and agronomic genes. Briefly, breeders first develop breeding objectives (e.g., yield), set up the requirements of new varieties for related agronomic traits, and input them into AI-GBB. Breeders then run AI-GBB with the AI-GBB database on a personal computer to identify parents that have the most desirable contents of yield and agronomic genes to meet the designed breeding objectives and requirements of new varieties for related agronomic traits. The identification of breeding parents is based on the favorable alleles and heterotic genotypes of the genes controlling breeding objectives and related agronomic traits. Particularly, breeders select parents that are the most complementary in the objective and agronomic genes, and that are promising to yield progeny with more favorable alleles and heterotic genotypes than the current best variety used for production. According to the contents of the favorable allele and heterotic genotypes of the yield and agronomic genes, AI-GBB helps breeders to design crosses between the selected parents to maximally merge their favorable alleles and heterotic genotypes into progeny. Finally, breeders make the designed crosses and generate F_1_ hybrid seeds in a greenhouse or an off-season nursery. If breeders aim to develop hybrid varieties, the hybrid seeds are directly used for multi-location variety testing to determine where the new variety is released for production. This is because a hybrid containing the most desirable combination of objective and related agronomic genes will perform best when grown in the environment to which it is best adapted. If breeders want to develop pure-line varieties, the F_1_ plants are self-crossed to generate F_2_ plants and subjected to progeny selection for the designed new variety according to the contents of their objective and agronomic genes, starting from F_2_ generation in the greenhouse. Because it has already been designed and its genotypes of objective and related agronomic genes are known, the new variety that is homozygous for favorable alleles of additive, partial, or complete dominant genes and heterozygous or homozygous for favorable alleles of over-dominant genes is identified and validated by performance prediction using the yield and agronomic genes by running AI-GBB. For example, if the breeding objective is to develop high-yielding varieties by merging the favorable alleles and heterotic genotypes of an additional six yield genes, a progeny plant that is homozygous for five of the six yield genes with additive, partial, or complete dominant effects, and heterozygous for one of them with over-dominant effects or exhibiting heterosis, could be identified at F_2_ generation at a rate of 1 plant per 2048 F_2_ seedlings. Breeders generate seeds for the new pure-line variety in a greenhouse or an off-season nursery and send them for multi-location variety testing to determine where the new variety can be released for production [2].

Three datasets of agronomic genes can be used for AI-GBB: (1) The total number of favorable alleles (total NFAs) of agronomic genes controlling each objective or related agronomic trait, including those exhibiting heterosis when heterozygous [5,6]; (2) the SNPs and/or InDels of the yield or agronomic genes significantly influencing trait performance (defined hereafter as functional SNPs/InDels); and/or (3) the expressions of their transcripts or splices responsible for the performance of the objective or related agronomic trait [3,5]. Liu et al. [5,6] tested the utility and efficiency of AI-GBB for pure-line varieties in cotton using cotton (*Gossypium* sp.) fiber length (*GFL*) genes and developed AI-GBB in cotton (CottonAI-GBB). Zhang et al. [3,4] tested the utility and efficiency of AI-GBB for inbred lines [3] and hybrid varieties [4] in maize using maize (*Zea mays*) inbred line grain yield (*ZmINGY*) and F_1_ hybrid grain yield (*ZmF1GY*) genes, respectively, and developed AI-GBB in maize (MaizeAI-GBB). For progeny phenotype prediction and selection, any of these three datasets of agronomic genes could accurately predict the performance of a targeted trait, with a prediction accuracy–Pearson’s correlation coefficient (*r*) between predicted and observed phenotypes of 0.8 or higher when 150 or more functional SNPs of the agronomic genes controlling the targeted trait were used [3,4,5,6,7]. These prediction accuracies surpassed those of genomic selection using up to 15,177 SNPs by 12–165% for cotton fiber length [6,8,9,10], up to 235,265 SNPs by 3–176% for maize inbred line grain yield [3], and up to 122,029 transcripts or 37,392 SNPs by 27–406% for maize F_1_ hybrid grain yield prediction from parents [4]. The number of agronomic gene functional SNPs was only 0.1–1.0% of those used for genomic prediction. Moreover, Liu et al. [5] and Zhang et al. [3] showed when the phenotypes of the objective or related agronomic traits predicted with two or all three datasets of agronomic genes were used for progeny selection, the best-performing plants selected with AI-GBB were up to 100% consistent with those selected based on plant performance determined by replicated field trials.

The aims of this study were to test whether the genes controlling an agronomic trait identified using a biparental population phenotyped in a single environment can be used for AI-GBB using the cotton *GFL* genes and to identify those applicable for AI-GBB across diverse populations, environments and eco-agricultural systems. The results of this research will dramatically promote the research, development, and implementation of AI-GBB in different field crops, vegetable crops, fruit trees, and livestock. Cotton, including upland cotton (*Gossypium hirsutum* L.) and Sea Island or Pima cotton (*G. barbadense* L.), is the global leading crop of natural textile fibers, producing approximately 30 million tons of lint and 50 million tons of oilseed annually [11]. Upland cotton produces more than 95% of the world’s cotton, while Sea Island cotton contributes less than 5% of the global cotton. The increasing demand for high-quality fibers, particularly long-staple types for their durability and spinnability, has made improvement in fiber length a central focus of contemporary cotton breeding [12,13].

To develop AI-GBB in cotton for high fiber quality, Liu et al. [5,6,7] previously identified 474 *GFL* genes (GenBank acc. No.: MW082098-MW082571) and evaluated their utility and efficiency for AI-GBB using a biparental population across two years at a single location in cotton. These *GFL* genes were identified from a recombinant inbred line (RIL) breeding population derived from an interspecific cross between upland cotton and Sea Island cotton. The functions of the 474 *GFL* genes were confirmed using several methods widely used for gene function research, including gene expression analysis, mutation analysis, genetic transformation, and phenotype prediction [5,6]. The utility and efficiency of the *GFL* genes for AI-GBB, especially their ability and efficiency in predicting fiber length, were tested with a biparental population at a single location using their functional SNPs/InDels [5], transcript expressions [5], and their NFAs [6,7], respectively, within and across years or generations, revealing high accuracy and consistency in fiber length prediction. In the present study, we tested the utility and efficiency of the *GFL* genes for AI-GBB across diverse populations and 11 environments and identified a set of *GFL* genes applicable for AI-GBB under any of the populations and environments tested. We further characterized the *GFL* genes applicable for AI-GBB across these conditions, providing a molecular basis for the development and deployment of AI-GBB in cotton, as well as probably in many other agricultural species, including field crops, vegetable crops, fruit trees, and livestock.

## 2. Results

### 2.1. Variation in Fiber Length

**Figure 1:**
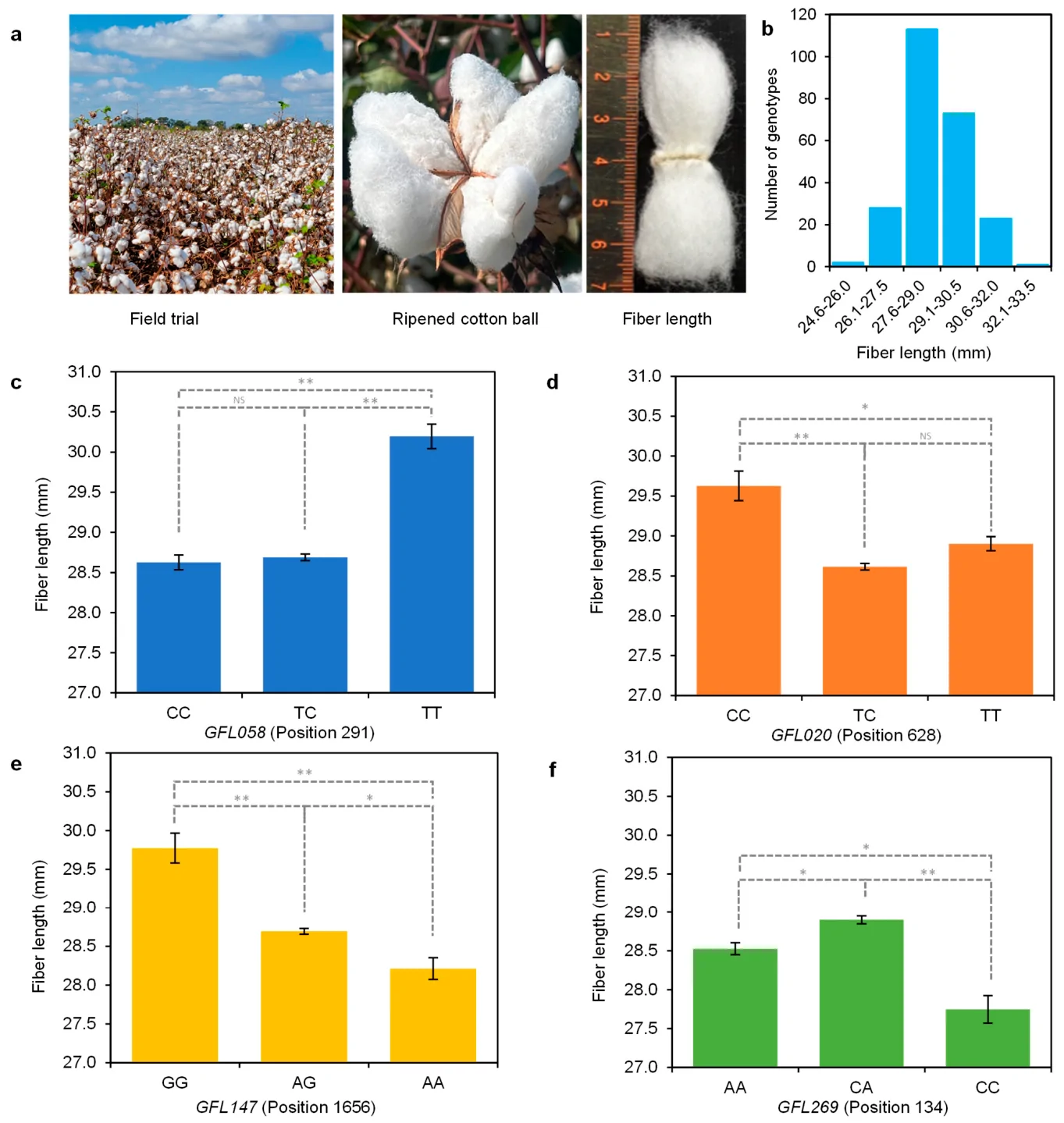
Examples of the nucleotide mutations of the 143 *GFL* genes and their impacts on fiber length in the upland cotton GWAS panel in China. (**a**) Field trial, ripened cotton ball, and fiber length. (**b**) The best linear unbiased prediction (BLUP) values of fiber lengths across all three years (2007, 2008, and 2009) and three locations (Anyang, Kuche, and Nanjing). (**c**) A SNP mutation from Nucleotide T to C at Position 291 of *GFL058* led to a decrease in fiber length by 5.48% from 30.20 mm to 28.63 mm when the mutation was homozygous, while the heterozygote of the mutation had a negative complete dominant effect on fiber length. (**d**) A SNP mutation from T to C at Position 628 of *GFL020* led to an increase in fiber length by 2.53% from 28.90 mm to 29.63 mm when the mutation was homozygous, while the heterozygote of the mutation had a negative over-dominant effect on fiber length. Although the fiber length of the heterozygote T/C group was not significantly shorter than that of the homozygote T/T group (*p* > 0.05), the difference was larger than 1.00% (see Table S6). (**e**) A SNP mutation from A to G at Position 1656 of *GFL147* led to an increase in fiber length by 5.53% from 28.21 mm to 29.77 mm when the mutation was homozygous, while the heterozygote of the mutation had an additive effect on fiber length. (**f**) A SNP mutation from C to A at Position 134 of *GFL269* led to an increase in fiber length by 2.81% from 27.75 mm to 28.53 mm when the mutation was homozygous, while the heterozygote of the mutation had an over-dominant effect on fiber length. Asterisks “*” and “**” indicate that the fiber lengths are different at *p* ≤ 0.05 and *p* ≤ 0.01, respectively. “NS” indicates that the difference in the fiber lengths is not significant at *p* ≤ 0.05. Error bar, standard error (SE).

An upland cotton genome-wide association study (GWAS) panel [14] and an interspecific breeding population and its two parents, designated as the TN population, were phenotyped for their fiber lengths across 11 environments (Figure 1a). The upland cotton GWAS panel consisted of 254 cultivars and landraces (Table S1) and their fiber lengths (Figure 1a,b), presented as upper half mean length (UHML), were phenotyped by three replicated field trials across Anyang, Kuche, and Nanjing in China, from 2007 to 2009 [14]. Fiber lengths exhibited a normal or nearly normal distribution in the panel across all three locations and all three years (Figure S1). Fiber lengths ranged from 23.2 mm to 33.9 mm, with an average of 28.7 mm to 29.2 mm and a coefficient of variance (CV) of 4.8–5.2%. The performance of fiber length was highly reproducible, with a correlation coefficient (*r*) of 0.73–0.88 (*p* < 0.0001) between years within each location (Figure S2), and 0.81–0.84 (*p* < 0.0001) between locations (Figure S3). We calculated the best linear unbiased prediction (BLUP) values of fiber length across all three years and three locations. The BLUP values of fiber lengths also showed a nearly normal distribution, ranging from 23.5 mm to 32.7 mm, with an average of 28.7 mm and a CV of 4.2% (Figure 1b).

The TN population consisted of 198 recombinant inbred lines (RILs) at F_8_ and F_9_ generations and their two parents. Fiber lengths, presented as UHML, of the TN population were phenotyped with three-replicated field trials in College Station, TX, USA in 2010 and 2011 [5]. Fiber length exhibited a normal or nearly normal distribution in both years (Figure S4). Fiber lengths ranged from 23.1 mm to 35.8 mm, with mean lengths of 28.5 mm and 28.7 mm and CV of 6.7% and 7.1%, respectively [5,6].

### 2.2. Nucleotide Sequence Variation in GFL Genes and Their Effects on Fiber Length

We examined the nucleotide sequence variation in the 474 *GFL* genes in the upland cotton GWAS panel. Of the 474 *GFL* genes, 461 (97.3%) contained at least one SNP, whereas 13 had no SNP in the panel. We identified a total of 14,940 SNPs in the 461 *GFL* genes, of which 475 SNPs contained in 143 (31%) of the 461 *GFL* genes affected fiber length BLUP values across all nine environments in China (*p* < 0.05) and were, therefore, considered as functional SNPs. Each functional SNP explained 1.0–7.0% of the fiber length variance (*p* < 0.05; Tables S2 and S3). Although the remaining 318 (69%) of the 461 *GFL* genes contained SNPs, none of them had significant impacts on the fiber length BLUP values (*p* > 0.05).

Given that cotton is a frequently outcrossing species, with an outcrossing percentage of 5–50%, depending on environments, heterozygous genotypes were identified for all 475 functional SNPs in the upland cotton GWAS panel. Among these functional SNPs, 85.1% affected fiber length BLUP values when they were present in homozygous form (*p* < 0.05); 84.6% affected fiber length BLUP values when they were present in heterozygous form (*p* < 0.05), and 46.3% had effects on fiber length when they were present in either homozygous or heterozygous form (*p* < 0.05) (Table S2). Of the functional heterozygous SNPs, 48.0% had negative impacts on fiber length, while 36.6% had positive impacts. Figure 1 shows four examples illustrating the nucleotide sequence variants of the 143 *GFL* genes and their effects on fiber length BLUP values in the upland cotton GWAS panel. The nucleotide substitutions in *GFL058*, *GFL020*, *GFL147*, and *GFL269* significantly altered fiber length BLUP values when they were in a homozygous state (*p* < 0.05), either increasing or decreasing fiber length. In a heterozygous state, however, the nucleotide substitutions exhibited diverse genetic effects on fiber length BLUP values, including negative complete dominance (Figure 1c), negative over-dominance (Figure 1d), additive (Figure 1e), and over-dominance (Figure 1f).

### 2.3. Ability and Efficiency of the 143 Functional SNP-Containing GFL Genes to Predict Fiber Length Performance

**Figure 2:**
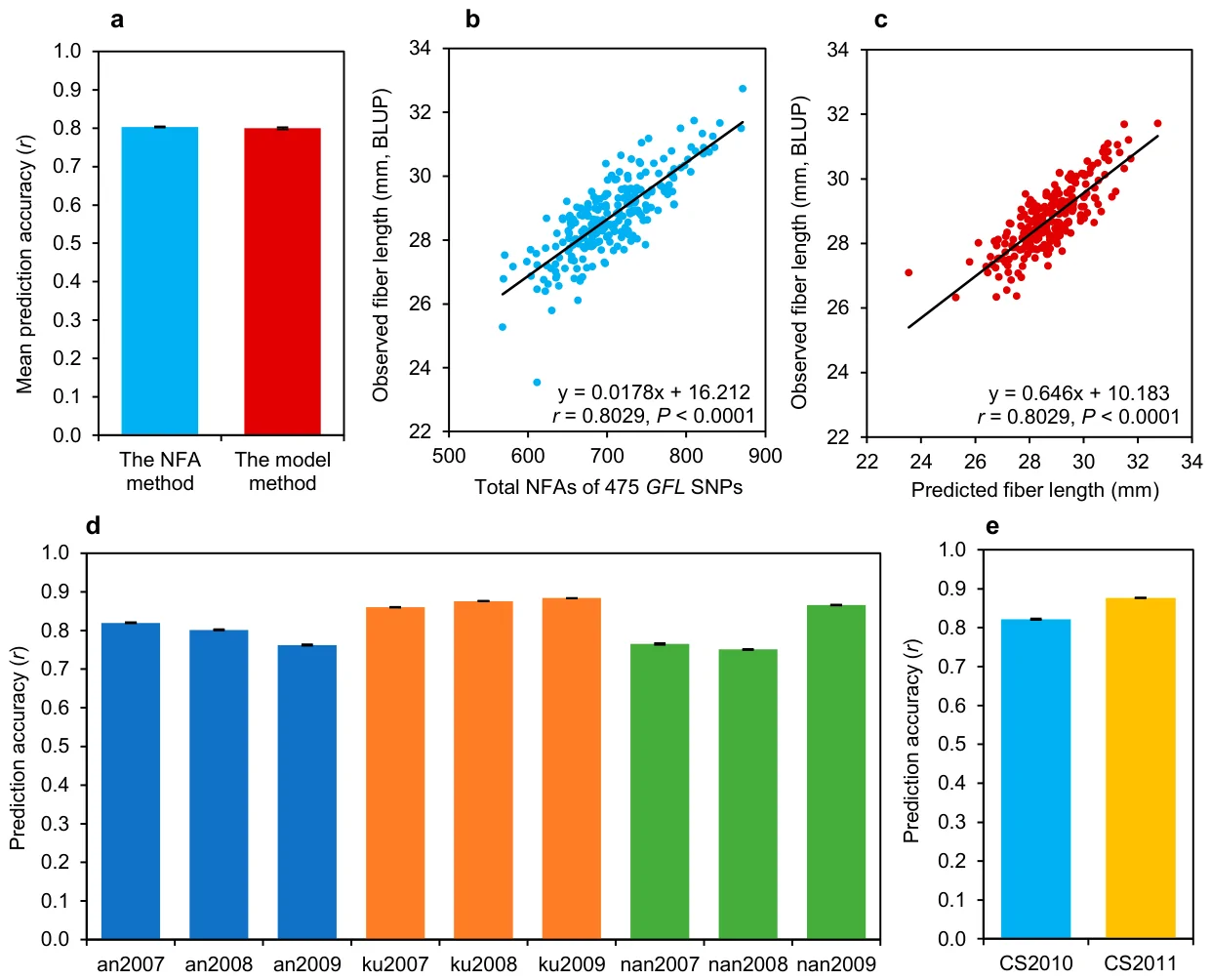
Ability and efficiency of the 143 functional SNP-containing *GFL* genes to predict fiber length of the 254 upland cotton GWAS panel cultivars and landraces (**a**–**d**) and the 198 TN population RILs (**e**). The total NFAs of the 475 functional SNPs contained in the 143 *GFL* genes and the BLUP values of fiber lengths across three years and locations/eco-agricultural systems in China were used for this experiment. (**a**) Prediction of fiber lengths with the 143 *GFL* genes using the NFA method and the model method, respectively. One hundred bootstrap replications were performed for the NFA method, and 100 replications with a five-fold cross-validation scheme were carried out for the model method. (**b**) Correlations between the total NFAs of the 475 SNPs contained in the 143 *GFL* genes and the BLUP values of fiber lengths determined by replicated field trials with the NFA method. (**c**) Correlations between the BLUP values of fiber lengths determined by replicated field trials and the fiber lengths predicted with the total NFAs of the 475 SNPs of the 143 *GFL* genes with the model method. (**d**) Prediction accuracies of fiber length based on the total NFAs of the 389–581 functional SNPs contained in 139–161 *GFL* genes in the upland cotton GWAS panel across years in Anyang (an2007, an2008, an2009), Kuche (ku2007, ku2008, ku2009) and Nanjing (nan2007, nan2008, nan2009), using the model shown in (**b; also see the model (1)**). (**e**) Prediction accuracies of fiber length based on the total NFAs of the 247 and 427 functional SNPs contained in the 113 and 160 *GFL* genes in the TN population in College Station, TX, USA in 2010 (CS2010) and 2011 (CS2011), respectively, using the model shown in (**b**). Error bar, standard error (SE).

We tested the ability and efficiency of the 143 *GFL* genes containing 475 functional SNPs to predict fiber length in the upland cotton GWAS panel using the NFA method and the model method [6], respectively. We calculated the total NFAs of functional SNPs across the 143 *GFL* genes in each cultivar or landrace (see Table S6) and predicted their fiber length BLUP values. The 143 *GFL* genes predicted fiber lengths of the 254 upland cotton diverse cultivars and landraces at high accuracy of 0.803 (*p* < 0.0001) using the NFA method and 0.800 (*p* < 0.0001) using the model method (Figure 2a). Figure 2b,c shows the relationships between the total NFAs of the 475 *GFL* functional SNPs and fiber length BLUP values, and between the predicted and observed fiber length BLUP values, with a Pearson’s correlation coefficient of *r* = 0.803 (*p* < 0.0001) for both. These results indicate that the 143 *GFL* genes can accurately predict fiber length across diverse upland cotton cultivars and landraces. The NFA method generated a fiber length prediction model (Figure 2b):

*y* = 0.0178*x* + 16.212 (1)

where *y* indicates the predicted fiber length BLUP value of a cultivar or landrace, and *x* indicates the total NFAs of *GFL* functional SNPs in that cultivar or landrace.

We next evaluated the utility and robustness of the prediction model (1) trained and validated above for fiber length prediction across years, locations, and eco-agricultural systems in China and the USA. We re-examined the SNPs contained in 461 *GFL* genes using the three-replication mean fiber lengths of the upland cotton GWAS panel determined in each year at each location in China. We found that 139–161 of the 461 *GFL* genes contained 389–581 functional SNPs in each environment. Among these *GFL* genes, 76–88% were shared with the 143 *GFL* genes identified using the fiber length BLUP values, while 12–24% were environment-specific, likely reflecting genotype × environment (G × E) interactions. This result suggested that a substantial number of the functional SNP-containing *GFL* genes identified in an environment could be applicable to AI-GBB in other environments. We predicted the fiber lengths of the upland cotton GWAS panel cultivars and landraces for each year at each location using the above prediction model (1). Prediction accuracies ranged from 0.75 to 0.88 (*p* < 0.0001) across years and locations (Figure 2d). For the TN population, we identified 247 functional SNPs contained in 113 of the 474 *GFL* genes for the 2010 trial and the 427 functional SNPs in 160 of the *GFL* genes for the 2011 trial. The differences in the numbers of *GFL* genes and functional SNPs between two years could also be attributed to the variation in *GFL* gene expressions and fiber lengths between the two years (see Figure S5). Of these 113 and 160 *GFL* genes, 61 and 74, respectively, were included in the 143 functional SNP-containing *GFL* genes identified in the upland cotton GWAS panel. We predicted the fiber lengths of the TN population RILs and parents using the total NFAs of the 247 and 427 functional SNPs, respectively, and prediction model (1). The *GFL* functional SNPs predicted the fiber lengths of the TN-population RILs and parents at accuracies of 0.82 and 0.88 for the 2010 and 2011 trials, respectively (Figure 2e). These results demonstrate that the fiber length prediction model (1), trained using the total NFAs of the 475 functional SNPs contained in the 143 *GFL* genes and the fiber length BLUP values of the upland cotton GWAS panel, is well suited for AI-GBB across populations, environments, and eco-agricultural systems.

**Figure 3:**
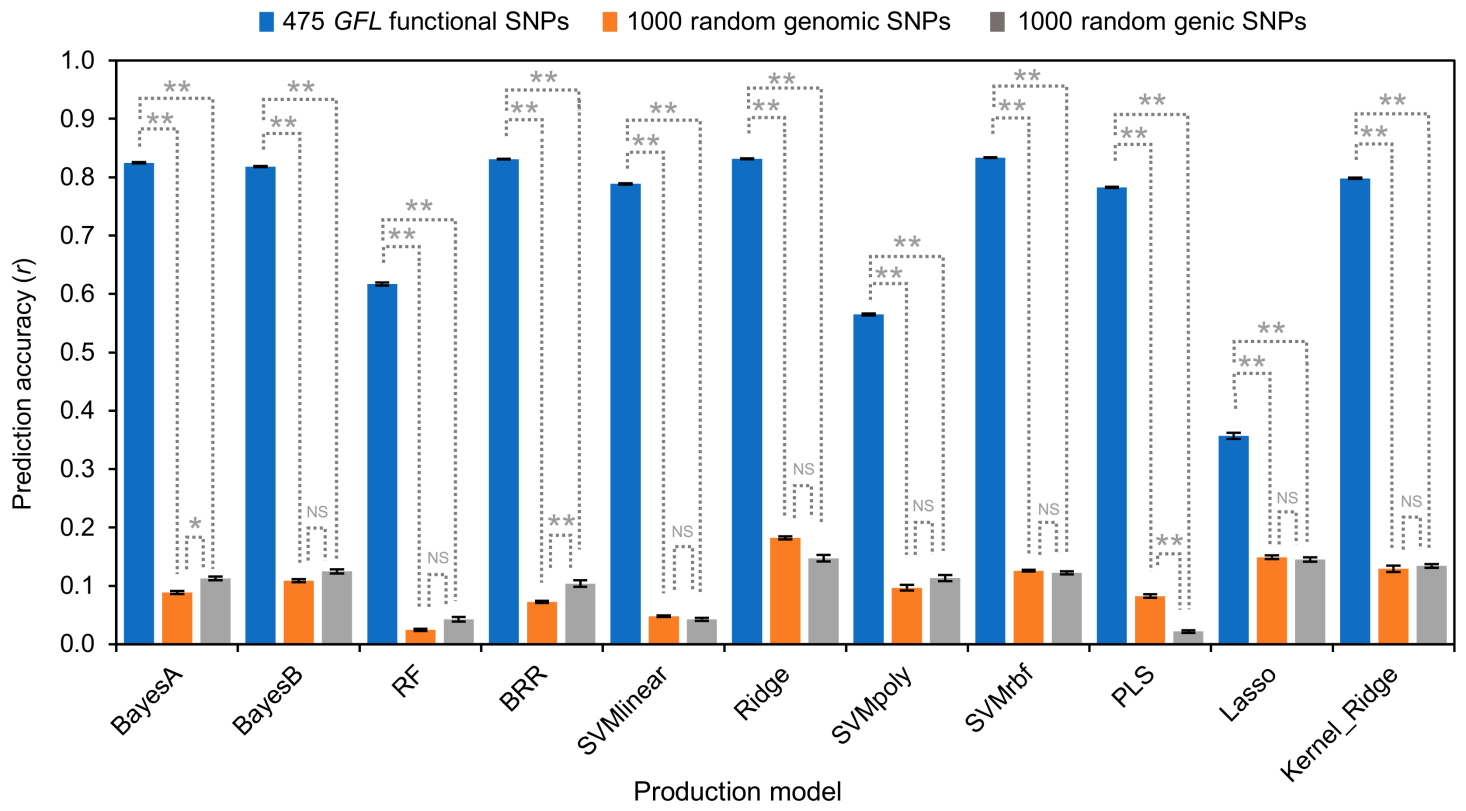
The 475 functional SNPs contained in the 143 *GFL* genes predicting the fiber lengths of the 254 upland cotton GWAS panel cultivars and landraces several-fold more accurately than 1000 genome-wide randomly sampled genomic or genic SNPs. The number of randomly sampled genomic or genic SNPs is over two-fold higher than those of the functional SNPs contained in the 143 *GFL* genes. The BLUP (best linear unbiased prediction) values of fiber lengths were used for the analysis. Asterisks “*” and “**” indicate that the prediction accuracies are different at *p* ≤ 0.05 and *p* ≤ 0.01, respectively. “NS” indicates that the difference in the fiber length prediction accuracies is not significant at *p* ≤ 0.05. Error bar, standard error (SE).

Furthermore, we compared the ability and efficiency of these 143 functional SNP-containing *GFL* genes with those of genomic selection using genome-wide randomly selected genomic and genic SNPs, respectively, for fiber length prediction in the upland cotton GWAS panel. Using the fiber length BLUP values as the fiber length phenotypes, we tested three SNP sets as predictors: (i) the 475 functional SNPs contained in the 143 *GFL* genes; (ii) 1000 genomic SNPs randomly sampled from the upland cotton GWAS panel genome-wide SNP dataset [14]; and (iii) 1000 genic SNPs randomly sampled from an upland cotton transcriptome-wide SNP dataset generated in this study. Eleven prediction models, including eight parametric and three non-parametric models, were used to test their prediction ability and efficiency. The 475 *GFL* functional SNPs predicted the fiber lengths of the upland cotton GWAS cultivars and landraces at accuracies ranging from 0.36 (Lasso) to 0.83 (SVMrbf, Ridge, and BRR) (Figure 3). In contrast, the 1000 random genomic SNPs failed to predict (*p* > 0.05) or successfully predicted (*r* ≥ 0.124, *p* ≤ 0.05) the fiber lengths of the upland cotton GWAS panel at accuracies of 0.02-0.18. Similarly, the 1000 random genic SNPs failed to predict or successfully predicted (*r* ≥ 0.124, *p* ≤ 0.05) the phenotypes of fiber lengths at accuracies of 0.02–0.15. The variation in fiber length prediction accuracies indicated that different prediction models differ in their ability to predict fiber length using either *GFL* gene functional SNPs or genome-wide randomly sampled SNPs, as reported previously [5]. The prediction accuracies of the 475 functional SNPs in the 143 *GFL* genes outperformed those of the 1000 random genomic or genic SNP set by several folds across all 11 prediction models tested.

**Figure 4:**
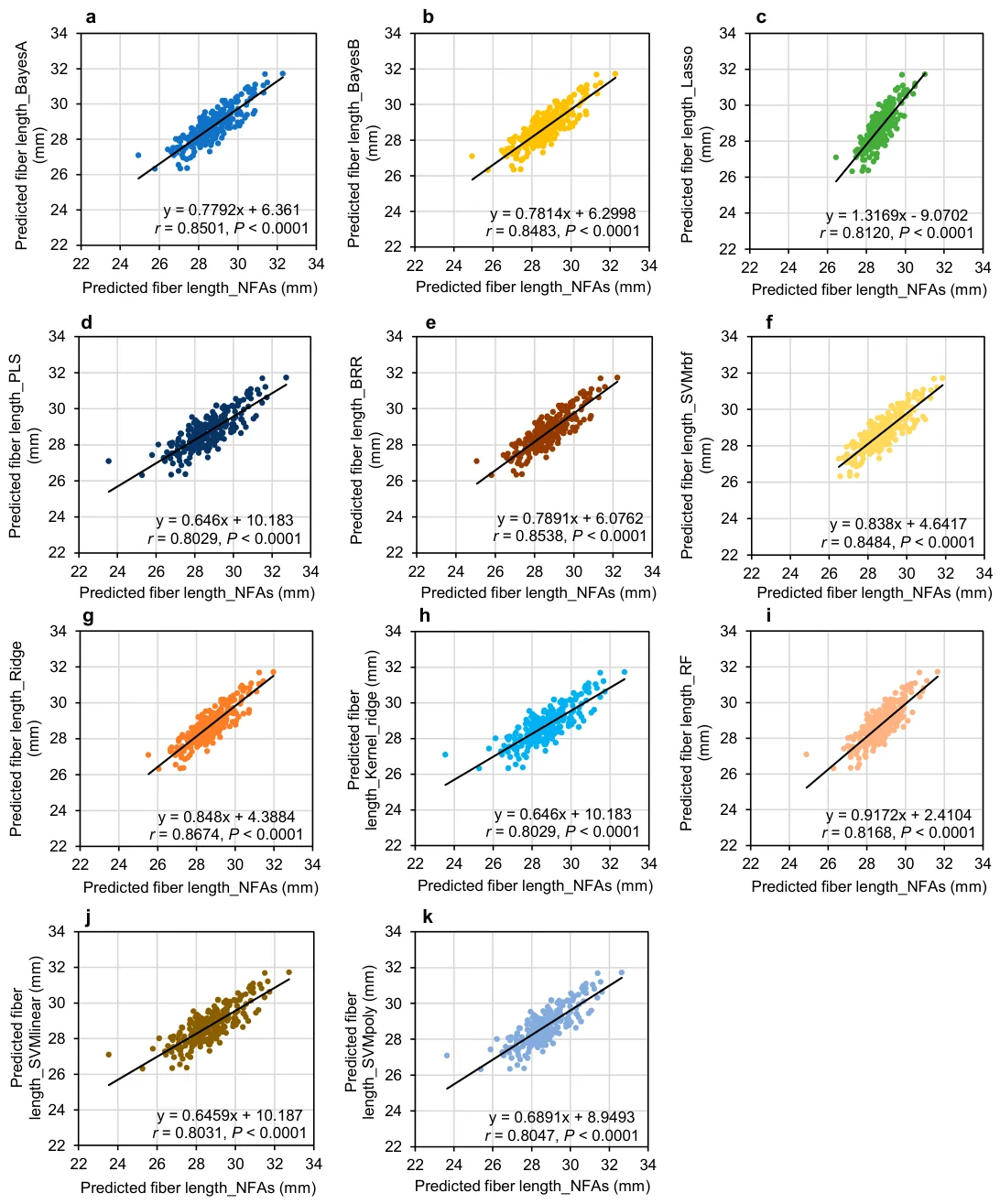
High correlations of predicted fiber lengths between the total NFAs and the markers of the 475 functional SNPs contained in the 143 *GFL* genes in the upland cotton GWAS panel. The fiber lengths predicted with the total NFAs of the 475 functional SNPs contained in the 143 *GFL* genes were compared with those predicted with the 475 functional SNPs of the 143 *GFL* genes as predictors using multiple regression models: (**a**) BayesA, (**b**) BayesB, (**c**) Lasso, (**d**) PLS, (**e**) BRR, (**f**) SVMrbf, (**g**) ridge, (**h**) Kernel_ridge, (**i**) RF, (**j**) SVMlinear, and (**k**) SVMpoly, respectively. For the full names of the prediction models, see the Materials and Methods Section. NFAs, number of favorable alleles.

Finally, we compared the fiber lengths of the upland cotton GWAS panel predicted using the total NFAs of the 475 functional SNPs of the 143 *GFL* genes with those predicted using the same 475 SNPs as markers with the 11 multiple regression models. The fiber lengths predicted with the 475 functional SNP NFAs were highly correlated with those predicted using the 475 SNPs as markers across all 11 tested multiple regression prediction models (*r* = 0.80–0.87, *p* < 0.0001) (Figure 4). These results indicate that the total NFAs of the 475 functional SNPs in the 143 *GFL* genes can serve as an effective and simpler marker system for accurate fiber length prediction, avoiding the complexity and computational burden associated with high-dimensional multiple regression models with a larger number of omic markers.

### 2.4. Applications of the 143 Functional SNP-Containing GFL Genes for AI-GBB

**Figure 5:**
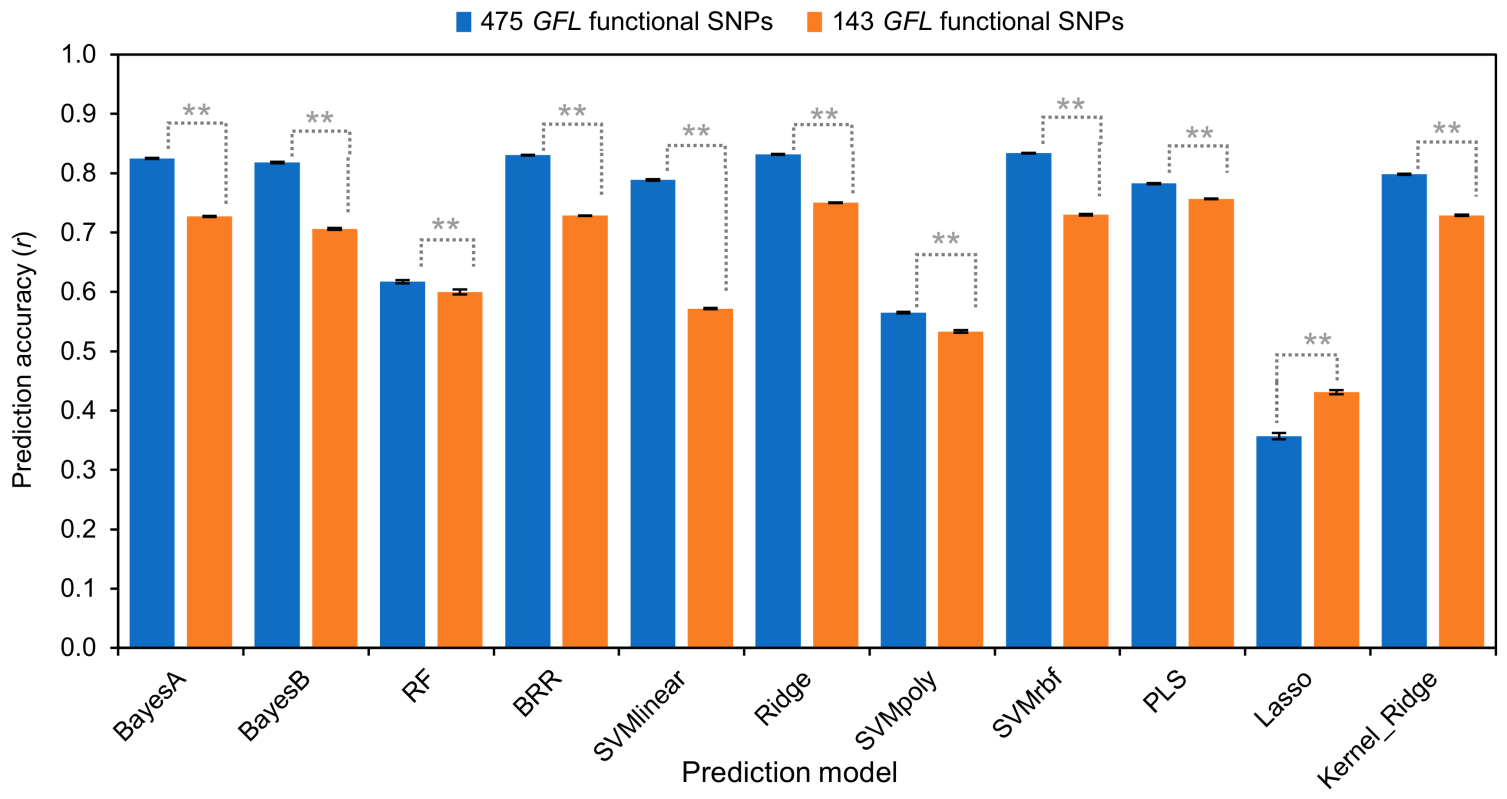
Prediction of fiber lengths in the upland cotton GWAS panel with 143 functional SNPs of the 143 *GFL* genes, with one functional SNP per *GFL* gene having the largest effect on fiber length versus their 475 functional SNPs. Asterisks “**” indicate that the difference in prediction accuracies is significant at *p* ≤ 0.01. Error bar, standard error (SE).

To facilitate application of the 143 *GFL* genes for AI-GBB, we tested whether one functional SNP per *GFL* gene for the 143 functional SNP-containing *GFL* genes would be sufficient for AI-GBB across populations, environments, and eco-agriculture systems. For each of the 143 *GFL* genes, we selected the functional SNP with the largest impact (highest *R^2^*) on fiber length BLUP values and tested the prediction accuracies of fiber length for the upland cotton GWAS cultivars and landraces using the 143 functional SNPs. The results were compared with those obtained using all 475 functional SNPs contained in the 143 *GFL* genes. The prediction accuracies of the 143 functional SNPs contained in the 143 *GFL* genes across the 11 prediction models ranged from 0.43 (Lasso) to 0.76 (PLS) (*p* < 0.0001) (Figure 5). The prediction accuracies of the 143 *GFL* functional SNPs were lower than those obtained with all 475 functional SNPs of the 143 *GFL* genes for 10 of the 11 prediction models tested (*p* < 0.001), except for the Lasso model, whose prediction accuracy was higher than that of 475 functional SNPs (*p* < 0.0001). Nevertheless, prediction accuracies of 0.70–0.76 were obtained with 143 *GFL* functional SNPs for 7 of the 11 prediction models (PLS, Ridge, SVMrbf, Kernel-ridge, BRR, BayesA, and BayesB) in descending order (Figure 5). These results demonstrate that using one functional SNP per *GFL* gene is also feasible for AI-GBB. Although the prediction accuracies of the 143 *GFL* functional SNPs, with one functional SNP per *GFL* gene, were lower than those predicted with all 475 functional SNPs of the *GFL* genes by 3.4–14.2%, the cost of genotyping breeding lines with the 143 *GFL* functional SNPs could be reduced by more than three-fold (475/143).

**Figure 6:**
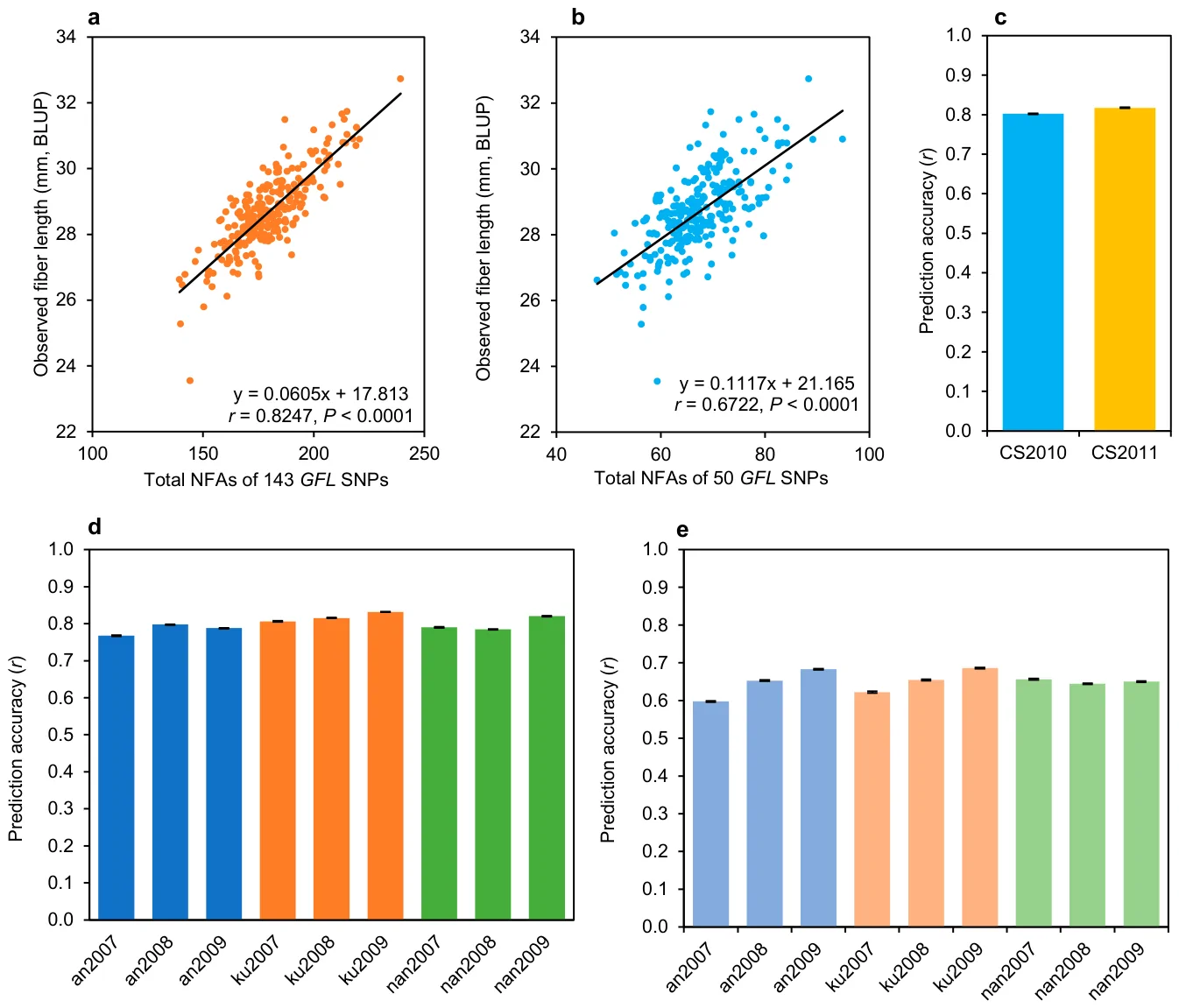
Prediction of fiber lengths across populations, environments, and eco-agriculture systems in China and the USA using the total NFAs of the 143 functional SNPs of the 143 *GFL* genes, with one SNP per *GFL* gene. (**a**) Pearson’s correlation coefficient, *r*, between the total NFAs of 143 functional SNPs contained in the 143 *GFL* genes and the fiber length BLUP values. (**b**) Pearson’s correlation coefficient, *r*, between the total NFAs of the 50 functional SNPs contained in the 50 *GFL* genes common across three years in Anyang, Kuche, and Nanjing and the fiber length BLUP values. (**c**) Prediction accuracies of fiber length based on the total NFAs of 61 and 74 functional SNPs contained in 61 and 74 of the 143 *GFL* genes identified in the TN breeding population in College Station, TX, USA, in 2010 (CS2010) and 2011 (CS2011), respectively, using the model trained in (**a; also see the model (2)**). (**d**) Prediction accuracies of fiber length in the upland cotton GWAS panel across years in Anyang (an2007, an2008, an2009), Kuche (ku2007, ku2008, ku2009) and Nanjing (nan2007, nan2008, nan2009) with the model trained (**a**) using 139–161 *GFL* genes whose SNPs had impacts on fiber length (*p* ≤ 0.05) in each environment. Of the 139–161 *GFL* genes, 109 (76%)–124 (88%) were contained in the 143 *GFL* genes identified above. (**e**) Prediction accuracies of fiber length in the upland cotton GWAS panel across years in Anyang (an2007, an2008, an2009), Kuche (ku2007, ku2008, ku2009) and Nanjing (nan2007, nan2008, nan2009) with the model trained in (**b; also see the model (3)**), using the total NFAs of 50 functional SNPs contained in 50 of the 143 *GFL* genes whose SNPs had impacts on fiber length (*p* ≤ 0.05) across all locations and all years. Error bar, standard error (SE).

To further evaluate the utility and efficiency of the 143 functional SNPs contained in the 143 *GFL* genes, we re-trained the prediction model with the total NFAs of the 143 functional SNPs and the fiber length BLUP values of the upland cotton GWAS panel. A prediction model with a prediction accuracy of *r* = 0.83 (*p* < 0.0001) (Figure 6a) was obtained:

*y* = 0.0605*x* + 17.813 (2)

We compared the 143 functional SNPs of the 143 *GFL* genes across individual years and locations in the upland cotton GWAS panel. We identified 50 of the 143 *GFL* genes that contained the same functional SNPs across all three years and three locations in the upland cotton GWAS panel (Table S4). We re-trained the prediction model with the 50 *GFL* functional SNPs and obtained the following prediction model, with a prediction accuracy of *r* = 0.67 (*p* < 0.0001) (Figure 6b):

*y* = 0.1117*x* + 21.165 (3)

We examined the ability and efficiency of the 143 *GFL* functional SNPs using the mean fiber length values of the TN population from the 2010 and 2011 trials, respectively, phenotyped at College Station (CS), TX, USA. We found that 61 and 74 of the 143 *GFL* genes contained functional SNPs in the CS2010 and CS2011 trials, respectively (Table S5). One functional SNP per *GFL* gene was selected and used to predict the fiber lengths of the TN population RILs and parents. The 61 and 74 functional SNPs of the 61 and 74 *GFL* genes predicted fiber lengths at accuracies of *r* = 0.80 and 0.82 (*p* < 0.0001) for the CS2010 and CS2011 trials, respectively (Figure 6c). These prediction accuracies were comparable to the accuracies of *r* = 0.82 and 0.88 obtained with the total NFAs of all 247 and 427 functional SNPs in the 113 and 160 *GFL* genes for the two years (Figure 2e).

We also selected one functional SNP per *GFL* gene identified in each year at each location, calculated their total NFAs, and predicted the fiber lengths of the upland cotton GWAS panel or the TN population using the prediction model (2), trained with the total NFAs of the 143 *GFL* functional SNPs and fiber length BLUP values, and the prediction model (3), trained with the total NFAs of the 50 *GFL* functional SNPs, respectively. The model trained with the 143 functional SNPs predicted fiber lengths in each year at each location at accuracies *r* = 0.77–0.83 (*p* < 0.0001) (Figure 6d), comparable to the accuracies of *r* = 0.75–0.88 obtained using the total NFAs of all 475 functional SNPs of the 143 *GFL* genes (Figure 2d). The prediction accuracies of the 50 *GFL* functional SNPs ranged from 0.60 to 0.69 across years at each location (Figure 6e).

**Figure 7:**
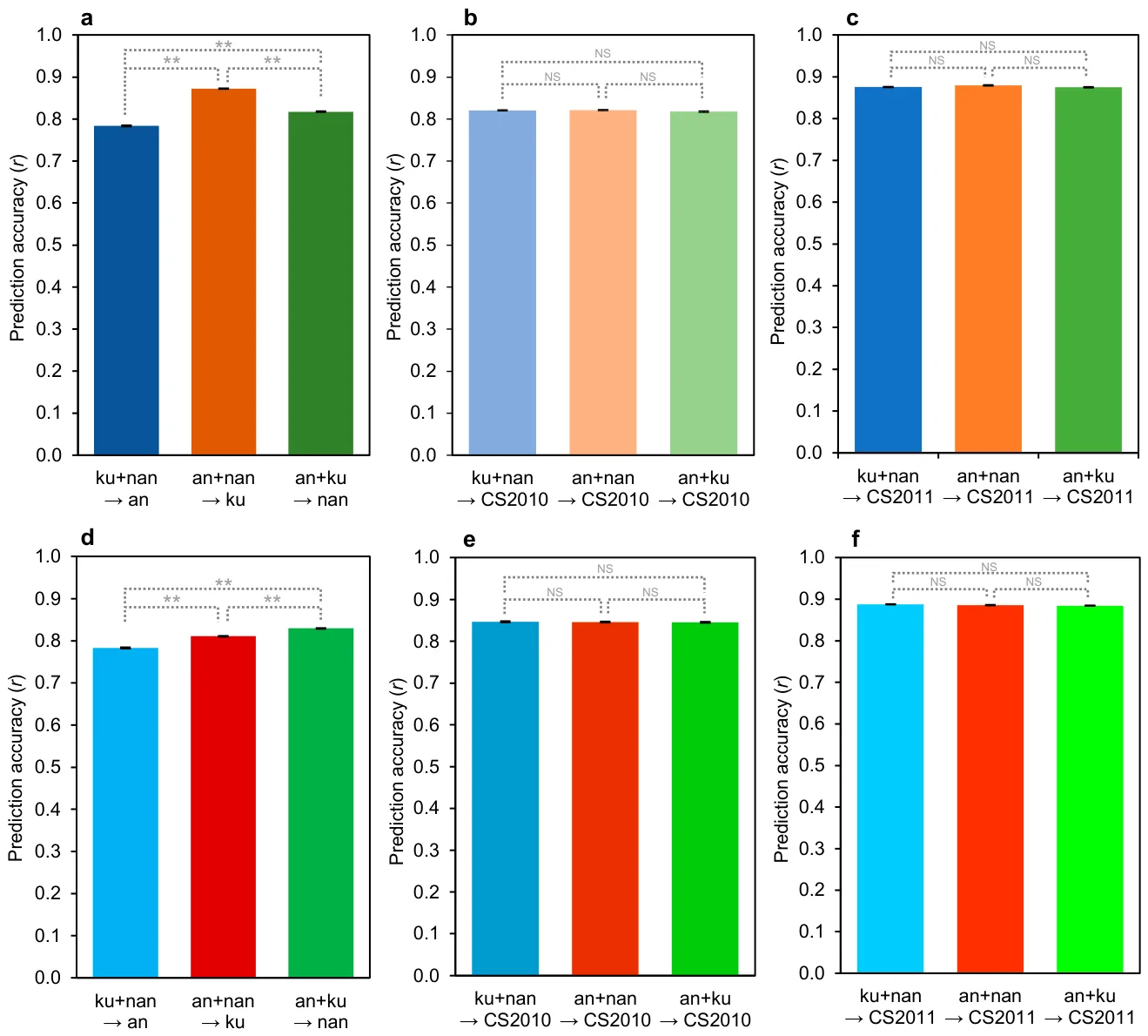
Predicting the fiber lengths of the 254 upland cotton GWAS panel cultivars and landraces and the 198 RILs and parents of the TN population in one environment using the model trained in other environments. (**a**) Prediction accuracies of fiber lengths in the upland cotton GWAS panel grown in Anyang (an), Kuche (ku), and Nanjing (nan) using the model trained with the datasets collected from ku+nan (459 functional SNPs of 143 *GFL* genes), an+nan (509 functional SNPs of 156 *GFL* genes), and an+ku (450 functional SNPs of 132 *GFL* genes), respectively. (**b**) Prediction accuracies of fiber lengths in the TN population in College Station, TX, USA, in 2010 (CS2010) using the models trained in (**a**). (**c**) Prediction accuracies of fiber length in the TN breeding population in College Station, TX, USA, in 2011 (CS2011) using the models trained in (**a**). (**d**) Prediction accuracies of fiber length in the upland cotton GWAS panel in an, ku, and nan using the models trained with ku+nan (143 functional SNPs of the 143 *GFL* genes, with one SNP per gene), an+nan (156 functional SNPs of the 156 *GFL* genes), and an+ku (132 functional SNPs of the 132 *GFL* genes), respectively. (**e**) Prediction accuracies of fiber length in the TN breeding population in College Station, TX, USA, in 2010 (CS2010) using the models trained in (**d**). (**f**) Prediction accuracies of fiber length in the TN breeding population in College Station, TX, USA in 2011 (CS2011) using the models trained in (**d**). Asterisks “**” indicate that the prediction accuracies are different at *p* ≤ 0.01. “NS” indicates the difference in the fiber length prediction accuracies is not significant at *p* ≤ 0.05. Arrow indicates the prediction of fiber length in one environment using the prediction model trained in other environments, e.g., predicting the fiber length of the cultivars and landraces in Anyang (an) using the model trained in Kuche (ku) and Nanjing (nan). Error bar, standard error (SE).

Furthermore, we tested whether the fiber lengths of the upland cotton GWAS panel in one environment, defined as one location across three years, could be predicted using models trained in other environments. We re-examined the SNPs in 461 of the 474 *GFL* genes and identified 143 *GFL* genes with a total of 459 functional SNPs for the Kuche + Nanjing dataset, 156 *GFL* genes containing 509 functional SNPs for the Anyang + Nanjing dataset, and 132 *GFL* genes containing 450 functional SNPs for the Anyang + Kuche dataset. Prediction models were trained using the total NFAs of these functional SNP datasets and the fiber length BLUP values of Kuche + Nanjing locations, Anyang + Nanjing locations, and Anyang + Kuche locations, respectively. The models were then applied to predict the fiber lengths of the upland cotton GWAS cultivars and landraces at Anyang, Kuche, and Nanjing (Figure 7a), as well as those of the TN population in 2010 (Figure 7b) and 2011 (Figure 7c) at College Station, TX, USA. Across locations in China and years in the USA, prediction accuracies ranged from *r* = 0.78 to 0.88 (*p* < 0.0001) (Figure 7a–c).

Similarly, we trained prediction models with 143 *GFL* genes from the Kuche + Nanjing dataset, 156 *GFL* genes from the Anyang + Nanjing dataset, and 132 *GFL* genes from the Anyang + Kuche dataset, respectively, using one functional SNP per *GFL* gene. These models were then applied to predict the fiber lengths of the upland cotton GWAS panel at each location in China (Figure 7d) and in the USA in 2010 and 2011 (Figure 7e–f). Prediction accuracies ranged from *r* = 0.78 to 0.89 (*p* < 0.0001), comparable to those obtained with models using the total NFAs of all functional SNPs contained in the *GFL* genes (*r* = 0.75–0.88, *p* < 0.0001) (Figure 2d,e).

### 2.5. Characteristics of the 143 and 50 Functional SNP-Containing GFL Genes Selected for AI-GBB

The next question was what made the 143 and 50 *GFL* genes capable for accurate prediction of fiber length and, thus, AI-GBB across diverse populations, environments, and eco-agricultural systems in China and the USA, given the genetic background-by-gene interaction and G x E interaction. To answer this question, we conducted enrichment analysis of the 474, 143, and 50 *GFL* genes in the genes expressed across all 198 RILs and parents of the TN population, the number of genes with two or more transcripts per gene, GO subcategories, and KEGG pathways. Although all three sets of the 474, 143, and 50 *GFL* genes were enriched in both the genes and transcripts expressed across all 198 RILs and parents of the TN population (Figure S5a) and in the number of genes with two or more transcripts per gene (Figure S5b) (*p* < 0.01) relative to all 74,402 genes, the enrichments in 143 *GFL* genes relative to the 474 *GFL* genes and the 50 *GFL* genes relative to 143 *GFL* genes were increased, decreased, or not observed in the percentages of genes and transcripts.

**Figure 8:**
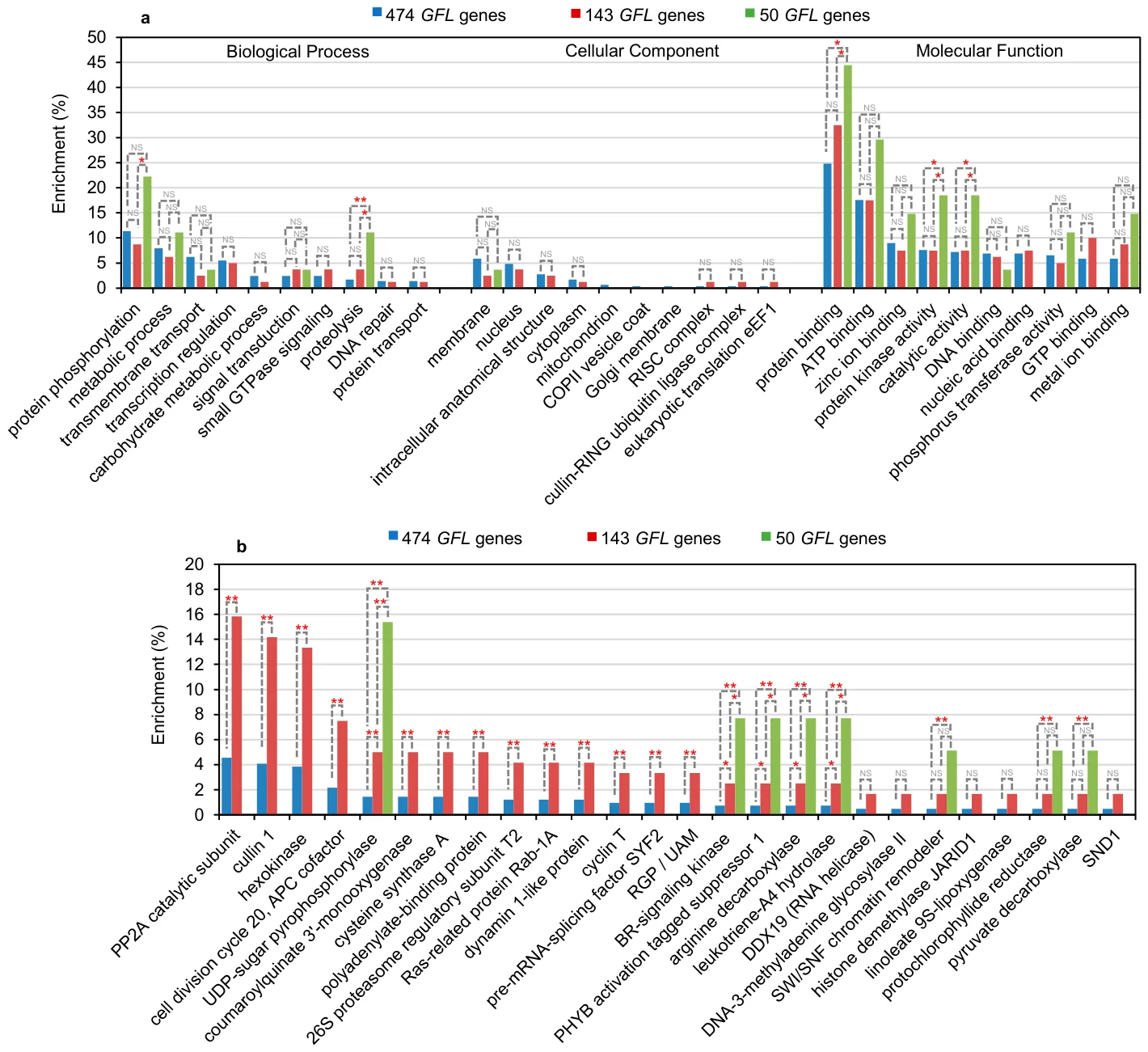
Examples of enrichment analysis results of gene ontology (GO) categorization (**a**) and KEGG pathway (**b**). For further details, see Supplementary Tables S7 and S8. For KEGG (Kyoto Encyclopedia of Genes and Genomes) pathways, see [15]. “*” and “**” indicate that the enrichment is significant at *p* ≤ 0.05 and *p* ≤ 0.01, respectively. “NS” indicates that the enrichment is not significant at *p* ≤ 0.05.

For GO categorization, the 474 *GFL* genes were categorized into 263 subcategories of the Biological Process (BP), Cellular Component (CC), and Molecular Function (MF) categories (Table S7). Of the 30 top GO subcategories further analyzed, five were enriched for the 474 *GFL* genes relative to all 74,402 genes, including BP metabolic process and transmembrane transport, CC RISC complex and acetyl–CoA carboxylase complex, and MF metal ion binding (Figure S6). The 143 and 50 *GFL* genes were categorized into 80 and 36 subcategories of the BP, CC, and MF categories, respectively (Table S7). Of the top 30 GO subcategories further analyzed, five were enriched for the 143 *GFL* genes relative to the 474 *GFL* genes and for the 50 *GFL* genes relative to the 474 and 143 *GFL* genes, including BP protein phosphorylation and proteolysis, and MF protein binding, protein kinase activity, and catalytic activity (Figure 8a).

The 474, 143, and 50 *GFL* genes were mapped to 146, 34, and 8 pathways, respectively (Table S8). Of the 26 top KEGG pathways further analyzed, all were enriched for the 474 *GFL* genes relative to all 74,402 genes (Table S6), 18 were enriched for the 143 *GFL* genes relative to the 474 *GFL* genes, 5 were enriched for the 50 *GFL* genes relative to both 474 and 143 *GFL* genes, and 3 were enriched for the 50 *GFL* genes relative to only the 474 *GFL* genes. The five enriched subcategories of the 50 *GFL* genes included UDP-sugar pyrophosphorylase, BR-signaling kinase, PHYB activation tagged suppressor 1, arginine decarboxylase, and leukotriene-A4 hydrolase (Figure 8b). These enrichments may play significant roles in making the 143 and 50 *GFL* genes capable of AI-GBB across diverse populations, environments, and eco-agricultural systems.

## 3. Discussion

AI-GBB can be a next-generation technology for enhanced plant and animal breeding. This study, for the first time, consistently shows that the genes identified with a biparental population phenotyped at a single location are well applicable for AI-GBB across diverse populations, environments and eco-agricultural systems in China and the USA. The 143 *GFL* genes identified in this study that are polymorphic in a nucleotide sequence within upland cotton have significant impacts on fiber length or contain functional SNPs that enable the prediction of fiber length across populations, environments, and eco-agricultural systems at accuracies ranging from 0.76 to 0.88 (Figure 2d). The *GFL* genes also predicted fiber length performance of diverse populations grown in one environment at accuracies of 0.78–0.89 using models trained in other environments, thus providing an additional advantage for enhancing breeding over genomic selection. These prediction accuracies for fiber length were several times higher than those of genomic selection, with 1000 genome-wide randomly sampled genomic or genic SNPs resulting from this study. The prediction accuracies of the *GFL* genes were 14–132% higher than those of genomic selection using up to 13,330 genomic SNPs reported thus far in cotton [8,9,10,16]. The number of SNPs used in this study for genomic selection was over two-fold as many as that of the 475 functional SNPs contained in the 143 *GFL* genes, while those of SNPs used in previous studies [8,9,10,16] were over 28-fold as many as those of the 143 *GFL* genes. Although the performance of a biological trait controlled by genes may be modulated by genetic background x gene interaction and genotype or gene x environment interaction, the utility and efficiency of the *GFL* genes for AI-GBB are expected because they are the primary determinants of fiber length, and fibers are universal for all cultivated cottons. As shown in Figure S5a, approximately 82% of the 474 *GFL* genes were expressed across all 198 RIL genotypes and two parents of the TN population. The results of these experiments support the findings of the present study, suggesting that this research may also be applicable to other agronomic traits in cotton and agronomic traits in other species.

Genes controlling yield, quality, biotic stress resistance, abiotic stress tolerance, and related agronomic traits are the foundation of all breeding methods, including traditional breeding, AI-GBB [2], genomic-selection- or genomics-assisted breeding, gene editing, gene silencing, genetic engineering, and marker-assisted selection [1]. In this study, we have identified 143 *GFL* genes (30% of the 474 *GFL* genes) that are polymorphic among the 254 upland cotton cultivars and landraces at 475 nucleotides significantly impacting fiber length, i.e., 475 functional SNPs, with an average of 3.3 functional SNPs per *GFL* gene, when the BLUP values of fiber length across all environments and eco-agricultural systems were used for the analysis. These 143 *GFL* genes included 50 *GFL* genes (35%) and 166 functional SNPs that consistently impacted fiber length of the upland cotton GWAS panel across every environment and every eco-agricultural system studied. The number of the functional SNP-containing *GFL* genes varied from 139 (29%) to 161 (34%), collectively containing 389–581 functional SNPs, with an average of 2.8–3.6 functional SNPs per *GFL* gene, when the analysis was conducted by individual year at each location. These variations could be largely attributed to gene x environment interactions leading to variations in both *GFL* gene expressions and fiber length phenotypes. The numbers of functional SNP-containing *GFL* genes and functional SNPs within the upland cotton GWAS panel are substantially lower than the 226 functional SNP-containing *GFL* genes (48%) and 692 functional SNPs and 48 InDels, with 3.1 functional SNPs per *GFL* gene, previously identified with an interspecific upland cotton x Sea Island cotton population [5]. This result reflects the lower diversity of the *GFL* genes within upland cotton species than between upland and Sea Island cotton species, suggesting that the genetic variation in fiber length between upland cotton and Sea Island cotton has tended to be driven largely by the number of functional SNP-containing *GFL* genes, whereas genetic variation in fiber length within upland cotton has tended to be driven largely by the number of functional SNPs in each *GFL* gene.

The *GFL* genes and functional SNPs identified in this study represent the first sets of fiber length genes and functional SNPs applicable for enhanced cotton breeding across diverse populations, environments, and eco-agricultural systems. They are not only applicable but also the most desirable for all breeding methods, including AI-GBB, conventional breeding, genomic-selection- or genomics-assisted breeding, gene editing, gene silencing, genetic-engineering, and marker-assisted selection [1]. First, plant breeding often includes parent selection, cross-design, and progeny selection [1,2]. Unlike genome-wide random SNPs that have been efficient for progeny genomic selection but whose efficiency for parent selection and cross-design in genomics-assisted breeding remains to be tested, the *GFL* genes and their functional SNPs are efficient not only for progeny selection, but also for parent selection and cross-design. For instance, parents that are the most desirable for achieving breeding objectives can be accurately identified, and crosses that can maximally merge the favorable alleles and heterotic genotypes of the *GFL* genes from the selected parents into progeny can be designed based on their functional SNPs, including favorable/unfavorable alleles and heterosis/non-heterosis genotypes (e.g., Figure 1). Therefore, the *GFL* genes and their functional SNPs make it possible to develop cotton varieties with longer fiber length by design with a computer in a factory production-like manner in a greenhouse or an off-season nursery. Importantly, parent selection is the most crucial to breeding success among the three breeding steps because it is impossible to identify and develop a new superior variety from a breeding progeny population without superior individuals, regardless of the accuracy of progeny selection [2]. Second, for progeny selection, the *GFL* genes and their functional SNPs predict fiber length performance at accuracies of 14% to several times higher than those of genomic selection with tens of thousands of genome-wide SNPs [8,9,10,16]. Zhang et al. [4] showed in maize that when the grain yield phenotypes predicted with the NFAs and the functional SNPs of the functional SNP-containing grain yield genes were jointly used for progeny selection, the top plants or lines selected with the functional SNPs and NFAs of the grain yield genes were up to 100% consistent with those selected based on grain yield determined by replicated field trials. Third, the use of approximately 150 functional SNPs, with one function SNP per gene, for progeny selection could reduce the cost of genotyping a breeding population by hundreds of times relative to the cost of genotyping for genomic selection that often employs tens of thousands of genome-wide SNPs. Fourth, because the new varieties to be developed are designed based on the contents of the *GFL* genes and their genotypes are known, they can be identified as early as F_2_ generation, thus dramatically accelerating the breeding process. For instance, if a breeding objective is to merge the favorable alleles and heterotic genotypes of six additional *GFL* genes to a new variety, the minimal size of the F_2_ population is 2048 plants, allowing one plant to be homozygous or fixed for the favorable alleles of five of the six *GFL* genes and heterozygous for one heterosis gene that is to be identified at F_2_ generation with the functional SNPs of the *GFL* genes. Fifth, because the nucleotide sequences and functional SNPs of the *GFL* genes are known, a *GFL* gene functional SNP array and/or a high-throughput and cost-efficient genotyping-by-*GFL* gene targeted sequencing method using the Illumina sequencing platform could be developed for AI-GBB. The method not only makes it possible to identify the designed progeny plant and develop into a new variety at earlier generation, but also greatly reduce the cost of AI-GBB. Finally, knowledge of the nucleotide sequences and functional SNPs of the *GFL* genes facilitates and enables the development of simple models, simple algorithms, and software for AI-GBB, which enables breeders to develop superior varieties automatically in a factory-production-like manner in a greenhouse.

The 143 *GFL* genes and their 475 functional SNPs also provide gene sources and genic tools for cotton breeding through all other modern molecular breeding methods developed to date. For gene editing, the *GFL* gene functional SNPs provide precise guidance on which *GFL* gene(s) it is to target, which nucleotides it is to modify, and how it edits alleles to further improve fiber length (e.g., Figure 1). For RNAi-mediated gene silencing and genetic engineering, the *GFL* genes are the choice of desirable fiber length genes for improving fiber length [5]. As DNA markers for marker-assisted selection, the advantages of functional SNPs are substantial. The functional SNPs contained in the fiber length genes minimize the potential genetic recombination between markers and casual genes, which frequently occurs in marker-assisted selection using locus flanking markers, making it more reliable and cost-efficient.

An agronomic gene database of most, if not all, cotton cultivars, breeding lines, and germplasm accessions used in different cotton breeding programs nation- or worldwide, including their nucleotide sequences, functional SNPs, favorable/unfavorable alleles, heterosis/non-heterosis genotypes, etc., is significant to efficiently practice AI-GBB. This study has initiated such a database of fiber length genes for AI-GBB in cotton and demonstrated that it is feasible to rapidly and readily extend the database to thousands of cotton cultivars and breeding lines using the existing genomic datasets, including those used for GWAS, TWAS (transcriptome-wide association study), pan-genome research, and other molecular studies. The database currently consists of 198 advanced breeding lines (RILs) and two parents, and 254 upland cotton cultivars and landraces genotyped with the 474 *GFL* genes. We previously identified 10,954 agronomic genes controlling different fiber traits in cotton, including the 474 *GFL* genes studied here, 1078 *GSTR* genes controlling fiber strength, 725 *GUNIF* genes controlling fiber uniformity, 742 *GELO* genes controlling fiber elongation, 881 *GMIC* genes controlling fiber micronaire or maturity and fineness, 3140 *GLY* genes controlling plot lint yield, 1420 *GLP* genes controlling lint percentage, and 2494 *GSY* genes controlling plot oilseed yield. According to Qi et al. [17], at least 3700 cotton cultivars, breeding lines, and germplasm accessions, including the 254 upland cotton cultivars and landraces used in this study, have been sequenced. Analysis of the *GFL* genes and other fiber trait genes with these sequences can rapidly extend the cotton agronomic trait database to more than 3000 cultivars and breeding lines. Moreover, tens of thousands of remaining cotton breeding lines and germplasm accessions can be rapidly and cost-efficiently (approximately $20 per genotype) genotyped with the agronomic genes using an Illumina sequencing platform and added to the agronomic gene database for the practice of AI-GBB in cotton.

Analysis of the *GFL* genes controlling fiber length, a typical quantitative trait, in this study within the TN population and the upland cotton GWAS panel provides new insights into the molecular mechanisms underlying quantitative traits. For instance, 29–34% and 48% of the observed genetic variation in cotton fiber length were contributed by *GFL* gene nucleotide substitution, while 66–71% and 52% were contributed by *GFL* gene expressions that could be regulated by RNA alternative splicing, gene × gene interaction or networks, epigenetic factors, and genotype × environment (G × E) interaction within upland cotton and between upland cotton and Sea Island cotton, respectively. The genes controlling a quantitative trait, such as the 474 *GFL* genes, are significantly enriched in the number of the genes expressed across genotypes and those alternatively spliced into two or more transcripts. This finding is consistent with those reported by [18]. Moreover, the genes controlling a trait across populations, environments, and eco-agricultural systems, such as the 50 *GFL* genes, tend to be enriched only in a limited number of GO terms and/or pathways.

## 4. Materials and Methods

### 4.1. Plant Materials

An upland cotton genome-wide association study (GWAS) panel [14] and an interspecific biparental breeding population [5], defined as the TN population, were used in this study. The upland cotton (*Gossypium hirsutum*) GWAS panel consisted of 254 diverse and representative upland cotton cultivars and landraces sourced from Africa, Asia, Brazil, Russia, the USA, and China (Table S1). The TN population consisted of its two parents and 198 recombinant inbred lines (RILs) at F_8_ (eighth filial), and F_9_ generations, derived from a cross between upland cotton (*G. hirsutum*) acc. TAM 94L-25 and Sea Island cotton (*G. barbadense*) acc. NMSI 1331.

### 4.2. Genes

The 474 *GFL* genes controlling fiber length, previously identified from a biparental population by our laboratory, were used in this study (NCBI GenBank accession numbers: MW082098-MW082571) [5]. The functions of the *GFL* genes were confirmed by four methods widely used for gene function research, including gene expression analysis, gene mutation analysis, genetic transformation, and fiber length phenotype prediction [5,6,7].

### 4.3. Phenotyping

The fiber length data of the 254 upland cotton GWAS panel cultivars and landraces were from Fang et al. [14]. They were phenotyped through three-replicated field trials across diverse environments, including Nanjing (32°03′39″ N, 118°46′44″ E), Anyang (36°05′56″ N, 114°23′31″ E), and Kuche (41°42′56″ N, 82°55′56″ E), China, in 2007, 2008, and 2009 [14]. Fiber length was presented as upper half mean length (UHML), measured by a High-Volume Instrument (HVI). The fiber length data of the 198 RILs and their parents in the TN population were obtained from Liu et al. [5]. They were phenotyped through three-replicated field trials at College Station (30°36′05″ N, 96°18′52″ W), TX, USA, in 2010 (F_8_) and 2011 (F_9_) and presented as UHML measured by HVI [5].

Moreover, we calculated the best linear unbiased predictions (BLUPs) of the fiber length across all three years and three locations for the upland cotton GWAS panel to account for environmental variation and obtain more accurate and representative fiber length phenotypic estimates. These fiber length estimates were determined using the Ime4 package in R with the following model:

Y = lmer(Trait ~ Year × Location + (1 | Line) + (1 | Line:Location), data = df)

where Y, Trait, Line, Year, and Location represent predicted fiber length, observed fiber length, genotype, years, and locations, respectively.

### 4.4. Genotyping

Fang et al. [14] previously re-sequenced the whole genomes of the upland cotton GWAS pane using the Illumina HiSeq 2500 platform (San Diego, CA, USA), with each cultivar or landrace having an average of 5 x genome coverage. We downloaded the sequence reads from the NCBI Sequence Read Archive under accession numbers SRP047301 and PRJNA375965 (SPR106507). Fang et al. [14] identified 1,871,401 high-quality SNPs (minor allele frequency ≥ 0.05, missing data rate < 0.20, and base calling quality ≥ Q20) in the GWAS panel. We randomly sampled 1000 genomic SNPs from the high-quality SNP dataset of the upland cotton GWAS panel [14] for this study.

We genotyped the genes of the upland cotton GWAS panel using SAMtools [19,20] v1.16 with the whole-genome sequencing reads of the panel [14], using the upland cotton acc. TAM 94L-25 10-dpa (10 days post-anthesis), developing fiber transcriptome [21] as the reference. A total of 931,428 genic SNPs were obtained for the upland cotton GWAS panel. The genic SNPs with a minor allele frequency < 0.05, missing data rate ≥ 0.20, and base calling quality < Q30 were filtered out, resulting in 125,653 high-quality genic SNPs. We randomly selected 1000 genic SNPs and extracted the genic SNPs of the 474 *GFL* genes from the genic SNP dataset of the upland cotton GWAS panel for this study. The genic SNPs of the TN population were previously generated in our laboratory, and the genic SNPs of the 474 *GFL* genes were extracted by Liu et al. [5].

### 4.5. Identification of the Functional SNPs Contained in GFL Genes That Have Significant Impacts on Fiber Length

Genes have two types of SNPs: synonymous SNPs that do not alter the amino acid sequence of a coding protein, and non-synonymous SNPs that alter the amino acid sequence of a coding protein. Therefore, a vast majority of synonymous SNPs have no significant impact on biological traits (*p* > 0.05), whereas most, if not all, non-synonymous SNPs have significant impacts on biological traits (*p* ≤ 0.05). It was previously estimated that 80–95% of the SNPs contained in a gene are synonymous, while 5–20% are non-synonymous [3,5,22].

We identified a functional SNP contained in a *GFL* gene by determining whether it had a significant impact on fiber length. Because all SNPs are biallelic, with three genotypes—two homozygotes (e.g., AA and aa) and one heterozygote (Aa)—we classified the upland cotton GWAS panel or the TN population into two (e.g., AA and aa, AA and Aa, or Aa and aa), or three groups (e.g., AA, Aa, and aa) based on the genotypes of an SNP contained in a *GFL* gene. The fiber lengths of the two or three groups were subjected to statistical analysis using Student’s *t*-test or one way-analysis of variance (one-way ANOVA). If the test showed that the fiber lengths of the two or three groups were significantly different at *p* ≤ 0.05, the SNP was considered a functional SNP of the *GFL* gene for further analysis.

### 4.6. NFA Counting

We counted the number of favorable alleles (NFAs) of a functional SNP contained in a *GFL* gene in a cultivar, landrace, or RIL and calculated the total NFAs of all or selected functional SNPs contained in all *GFL* genes under analysis, as described in Supplementary Table S6. First, the two homozygous groups (e.g., AA and aa) of a functional SNP contained in a *GFL* gene were identified in the upland cotton GWAS panel or the TN population, and their fiber lengths were subjected to Student’s *t*-test. If the mean fiber lengths differed between the two homozygous groups (*p* ≤ 0.05), for instance, “AA > aa”, then the “A” was classified as the favorable allele, while “a” was considered the unfavorable allele of the *GFL* gene. The SNP of the *GFL* gene was then considered as a functional SNP. In cases where only one of the two homozygous groups and the heterozygous group were present in the upland cotton GWAS panel or the TN population, the favorable and unfavorable alleles of the functional SNP were determined by comparing the phenotype mean of the homozygous group with the phenotype mean of the entire population. If the phenotype mean of the homozygous group was larger or smaller than the population mean, the allele was considered as favorable or unfavorable allele. The NFAs of the favorable allele homozygote of the functional SNP in a cultivar, landrace, or RIL (e.g., AA) were counted as “2”. The NFAs of the unfavorable allele homozygote of the functional SNP (e.g., aa) were counted as “0”.

When a cultivar, landrace, or RIL was heterozygous (e.g., Aa) for the functional SNP contained in the *GFL* gene, the NFAs of the functional SNP were determined based on its genetic effect. An NFA value of “−1” was counted for the “Aa” when the mean fiber length of the “Aa” group was more than 1% lower than that of the “aa” group, indicating a negatively over-dominant effect on fiber length. An NFA value of “0” was assigned to “Aa” when the mean fiber length of “Aa” was within ±1.0% of the “aa” group mean fiber length, reflecting a negatively complete dominant effect on fiber length. When the mean fiber length of “Aa” was greater than that of “aa” by over 1%, but less than that of “AA” by over 1.0%, it was considered an additive effect, and an NFA of “1” was assigned. If the mean fiber length of “Aa” was within ±1.0% of the “AA” mean fiber length, suggesting a complete dominant effect, its NFA was set to “2”. Finally, if the mean fiber length of “Aa” exceeded that of “AA” by more than 1.0%, defined as an over-dominant effect, an NFA of “3” was assigned. For example, see Figure 1c–f. If the functional SNP genotype of a cultivar, landrace, or RIL was missing or not available (NA), the mean NFAs of the functional SNPs having genotypic data in the cultivar, landrace, or RIL of the GWAS panel or the TN population were calculated and used to fill the gap to minimize or substantially reduce the bias introduced by missing data. Given that NFAs of the functional SNPs missing genotypic data are stochastic, −1, 0, 1, 2, or 3, filling in their NFAs with the mean NFAs of the SNPs having genotypic data would not, or would only minimally, alter the final total NFAs of all functional SNPs in a genotype, and therefore would not, or would only slightly, influence their phenotype prediction result. The total NFAs of all or selected functional SNPs of the *GFL* genes contained in the cultivar, landrace, or RIL were calculated by summing the NFAs of all or selected individual functional SNPs of the *GFL* genes.

### 4.7. Prediction of Fiber Length Using the Functional SNPs Contained in the GFL Genes

The utility and efficiency of the functional SNPs contained in the *GFL* genes for AI-GBB, especially for fiber length prediction across populations, environments, and eco-agricultural systems, were tested using their NFAs and SNPs as predictors, respectively. The NFA method and the model method were used, respectively, to predict the fiber length of the upland cotton GWAS panel cultivars and landraces or the TN population RILs with the total NFAs of the *GFL* functional SNPs, as previously described [5,6]. The NFA method directly uses the total NFAs of the functional SNPs to predict the fiber length of the GWAS panel or the TN population, with 100 bootstrapping replications on the cultivars and landraces, or RILs and parents. The model method employs the following simple linear regression model to predict the fiber length of the GWAS panel and TN population:

*y* = *β_0_* + *βx* + *ε*,

where *y* is the predicted fiber length of a cultivar, landrace, or RIL; *x* is the total NFAs of all functional SNPs contained in the cultivar, landrace, or RIL; *β_0_* is the regression intercept; and *β* is the regression slop; and *ε* is sampling error. A five-fold cross-validation scheme, with 100 replications, was implemented in R (RStudio 4.3.0).

The fiber lengths of the upland cotton GWAS panel or the TN population were predicted using the functional SNPs of the *GFL* genes as prediction markers, without consideration of their genetic effects on fiber length, with eight parametric multiple regression models and three non-parametric regression models. The eight parametric multiple regression models included BayesA [23], BayesB [23], Bayesian ridge regression (BRR) [24], support vector machine linear (SVMlinear) [25], Ridge [26], partial least-squares (PLS) [27], least absolute shrinkage and selection operator (Lasso) [28], and Kernel_ridge [29]. The three non-parametric multiple regression models included random forest (RF) [30], support vector machine using the polynomial kernel function (SVMploy) [25], and support vector machine using the radial basis function kernel (SVMrbf) [25]. A ten-fold cross-validation scheme was implemented in Python (Version 3.13), with 100 replications. For comparison, the same software, the same eleven prediction models, ten-fold cross-validation scheme, and 100 replications were used to predict the fiber lengths of the GWAS panel with 1000 genome-wide randomly sampled genomic SNPs, and 1000 randomly sampled genic SNPs, respectively.

### 4.8. Characterization of the GFL Genes Applicable to AI-GBB Across Populations, Environments, and Eco-Agricultural Systems

To determine what characteristics of the 143 and 50 *GFL* genes make them applicable to the accurate prediction of fiber length, and thus AI-GBB across populations, environments, and eco-agricultural systems relative to the 474 *GFL* genes, we first examined their expressions and number of transcripts per gene across all 198 RILs and two parents of the TN population. We re-assembled the transcripts of the 198 RILs and two parents using their RNA-seq reads previously generated in our laboratory from 10 dpa developing fibers [5] using Trinity [31,32] v2.15.0. We quantified the expression of individual genes and transcripts or isoforms in the developing fibers of each RIL and parent of the TN population using RSEM software [33], with the Trinity assembly of upland cotton acc. TAM 94L-25, a parent of the TN population, as the reference. The numbers and the percentages of genes and transcripts of all 74,402 genes, all 147,987 transcripts, and the 474, 143, and 50 *GFL* genes and transcripts expressed across the entire TN population and parents were counted, calculated, and subjected to enrichment analysis using a chi-square (ꭓ^2^) test, comparing the 474 *GFL* genes relative to all genes, the 143 *GFL* genes relative to the 474 *GFL* genes, and the 50 *GFL* genes relative to the 143 *GFL* genes.

Then, we categorized the gene ontology (GO) of all 74,402 genes and the 474, 143, and 50 *GFL* genes expressed in the 10 dpa developing fibers of upland cotton acc. TAM 94L-25 and mapped them to the Kyoto Encyclopedia of Genes and Genomes (KEGG) pathways [15]. We blasted all genes, 474 *GFL* genes, 143 *GFL* genes, and 50 *GFL* genes against the *G. hirsutum* TM-1 reference annotation, for which the GO categories and pathways of annotated genes are determined using CottonGen [34], and retained the best hit for each query gene sequence. The GO categories and KEGG pathways of all genes and the 474, 143, and 50 *GFL* genes were extracted from CottonGen *G. hirsutum* (AD1) “TM-1” genome NAU-NBI_v1.1 for further analysis.

The GO categories and KEGG pathways were subjected to enrichment analysis in R using the AnnotationDbi and KEGGREST packages. For each set or group of genes, the best BLAST hit and corresponding annotation were used to construct the gene-to-GO and gene-to-KEGG mapping tables. Counts of genes assigned to each GO term and KEGG pathway were summarized. To facilitate enrichment analysis among all genes, the 474 *GFL* genes, the 143 *GFL* genes, and the 50 *GFL* genes, percentage values of each gene set were calculated as the number of genes assigned to each category or pathway divided by the total number of annotated genes within each gene set. Categories were truncated for clarity and plot readability. To enable direct comparison among the gene sets, only categories present in at least one gene set were retained; missing values for any gene set were considered zero. The resulting annotation tables were used for high-level functional comparison and visualization across gene sets.

### 4.9. Statistical Analysis

One-way ANONA, Student’s *t*-test assuming unequal variances, and simple linear regression were conducted using R (RStudio 4.3.0) and Microsoft Excel 365. The *p*-values for all statistical analyses were presented at a two-tailed significance level.

## Data Availability

All datasets generated for this study are included in the article and/or Supplementary Materials. The nucleotide sequences of the 474 *GFL* genes can be found in NCBI GenBank under accession numbers MW082098-MW082571. The phenotypes of fiber lengths and genome sequencing reads of the upland cotton GWAS panel were from [14] and the phenotypes of fiber lengths and RNA-seq reads of the TN population were from [5].

## Supplementary Materials

The following supporting information can be downloaded at https://www.mdpi.com/article/10.3390/ijms27198908/s1.
